# Interleukin-25-mediated resistance against intestinal trematodes does not depend on the generation of Th2 responses

**DOI:** 10.1186/s13071-020-04467-7

**Published:** 2020-12-04

**Authors:** María Álvarez-Izquierdo, Miguel Pérez-Crespo, J. Guillermo Esteban, Carla Muñoz-Antoli, Rafael Toledo

**Affiliations:** Área de Parasitología, Departamento de Farmacia y Tecnología Farmacéutica y Parasitología, Facultad de Farmacia, Universitat de València, Avda. Vicent Andrés Estellés s/n, Burjassot, 46100 Valencia, Spain

**Keywords:** Interleuquin-25, Intestinal helminth, Th2, Resistance, Trematoda, Echinostoma caproni

## Abstract

**Background:**

The cytokine interleukin-25 (IL-25) is recognized as the most relevant initiator of protective T helper 2 (Th2) responses in intestinal helminth infections. This cytokine induces resistance against several species of intestinal helminths, including the trematode *Echinostoma caproni*. *E. caproni* has been extensively used as an experimental model to study the factors determining resistance to intestinal infections. In the study reported here, we assessed the role of IL-25 in the generation of resistance in mice infected with *E. caproni*.

**Methods:**

The factors that determine the production of IL-25 in mice experimentally infected with *E. caproni* were determined, as were the consequences of IL-25 production in terms of polarization of the immune response and resistance to infection.

**Results:**

Our results show that the role of IL-25 in the polarization of the immune response differs between the primary and secondary immune responses. IL-25 is required for the development of a Th2 phenotype in primary *E. caproni* infections, but it can also promote the differentiation to Th2 memory cell subsets that enhance type-2 immunity in memory responses. However, the development of Th2 responses does not induce resistance to infection. The Th2 phenotype does not elicit resistance, and IL-25 is responsible for the resistance regardless of its type-2 cytokine activity and activation of signal transducer and activator of transcription (STAT6). Alternative activation of macrophages induced by IL-25 can be implicated in the resistance to infection.

**Conclusions:**

In contrast to primary infection, secondary infection elicits a type-2 immune response even in the absence of IL-25 expression. Despite the development of a type-2 response, mice are susceptible to secondary infection associated with the lack of IL-25. Resistance to infection is due to the production of IL-25, which acts autonomously from Th2 response in terms of parasite clearance.
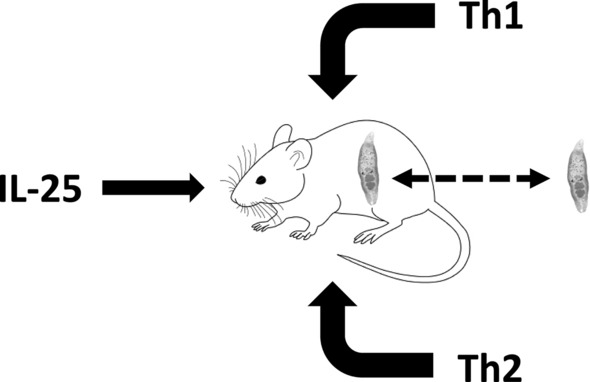

## Background

Intestinal helminth infections are common in humans and animals, especially in developing regions of Africa, Asia and the Americas [1–3]. In humans, these parasitic infections generate substantial morbidity and produce relevant physical and mental disorders that are often aggravated in the presence of serious economic problems [2]. In addition, infections by intestinal helminths compromise the health and productivity of livestock worldwide [3]. At the present time, the impact of intestinal helminth infections is primarily managed and reduced by treatment with anthelminthic drugs. However, the progressive emergence of resistance to these drugs limits their utility and, in addition, infections do not generate protective immunity, resulting in continuous reinfections in environments of poverty and poor sanitary conditions. To date, no effective vaccines have been developed to protect humans or animals from helminth infections. A major obstacle to successful immunization, among other factors, is the lack of knowledge on how protective immunity is selected after infection [4].

Resistance to intestinal helminths is based on the generation of T helper 2 (Th2)-cell responses in a complex process that involves the interaction between innate and adaptative mechanisms [5–7]. Protective Th2 immunity against intestinal helminths is initiated and amplified by the epithelial-derived alarmin cytokines, including interleukin (IL)-25, IL-33 and thymic stromal lymphopoietin (TSLP), although the immune mechanisms behind the development of these responses are poorly understood [6, 8]. In recent years, IL-25, a member of the IL-17 family of cytokines, also called IL-17E, has been considered to be a key cytokine since it promotes Th2 immunity and exerts anti-inflammatory functions via the downregulation of Th17 and Th1 responses [9–12]. IL-25 expression is generally associated with resistance to gastrointestinal helminth infections through the activation of Th2 responses that mediate effector mechanisms for parasite expulsion (which include goblet cell hyperplasia, smooth muscle hypercontractility, expression of resistin-like molecule-beta [RELM-β] and intestinal mastocytosis, among others) [6]. While recent studies have uncovered the origin and the mechanisms of action of IL-25 [13–15], several doubts remain concerning the role of IL-25 in the generation of protective Th2 responses to intestinal helminth infections [8, 16, 17]. For example, it is not well defined if the participation of IL-25 is limited to its ability to promote Th2 responses or if it is directly involved in the activation of effector mechanisms responsible for resistance. Likewise, its potential role in the differentiation of Th cells to memory subset cells and their implications in the generation of immunity against intestinal helminths is unknown. Several recent studies have questioned the role of IL-25 on the generation of adaptive type-2 immune responses or on the differentiation of Th2 cells or their development to effector or memory Th2-cell subsets [8, 16].

In addition to members of genus* Echinostoma* being of interest as human parasites, mainly in East and Southeast Asia [18, 19], they, and in particular *Echinostoma caproni* (Trematoda: Echinostomatidae), have been extensively used for the establishment of chronic infections or the development of resistance to intestinal helminths in experimental studies. *E. caproni* is an intestinal trematode with no tissue phase in the vertebrate definitive host [20]. After infection, the metacercariae excyst in the duodenum and the juvenile worms migrate to the ileum, where they attach to the mucosa. *E. caproni* has a wide range of definitive hosts, although its compatibility differs considerably between rodent species in terms of worm survival and development [21]. In mice and other hosts of high compatibility, the infection becomes chronic, while in hosts of low compatibility (e.g. rats), the worms are expelled from the body 2–4 weeks post-infection [22, 23]. The establishment of chronic infections in the ICR mice line is dependent upon a local Th1 response with elevated production of interferon gamma (IFN-γ) [24]. In contrast, the resistance to *E. caproni* infection in hosts of low compatibility is associated with the development of a local Th2 phenotype [24, 25]. Because of these characteristics, the *E. caproni*-rodent model is useful to elucidate several aspects of the host–parasite relationship in intestinal infections, such as the induction of distinct effector mechanisms and their effectiveness in parasite clearance. Recent studies by our group have shown that partial resistance against *E. caproni* secondary infections in ICR mice is developed after the chemotherapeutic cure of a primary infection and that innately produced IL-25 is crucial to establishing resistance. Susceptibility to primary infections was associated with low levels of intestinal IL-25 gene expression, while deworming via administration of praziquantel (PZQ) was accompanied by a steady increase in IL-25 expression and, in turn, by the onset of a Th2-type response that prevented the establishment of secondary infections [26, 27].

In the study reported here, we investigated the role of IL-25 in resistance to *E. caproni* infections in mice. Our results show that IL-25, but not the type-2 immune response, is required for resistance. However, IL-25 could have a role in the differentiation of the Th2 memory subset, facilitating Th2 responses to challenge infections. Susceptibility of mice to *E. caproni* infections relies on the fact that parasite compounds do not elicit IL-25 responses in mice, and the upregulation of this cytokine depends on external factors.

## Materials and methods

### Parasites, hosts and experimental primary and secondary infections

Encysted metacercariae of *E. caproni* were removed from the kidney and pericardial cavity of experimentally infected *Biomphalaria glabrata* snails and used to infect male ICR mice weighing 30–35 g by gastric gavage, establishing both primary and challenge infections (50 metacercariae each). Positivity of infection in each case was determined at necropsy or detection of eggs in stools [22]. Animals were maintained under conventional conditions with food and water ad libitum. Each experiment was performed in triplicate.

### Ethical statement

This study has been approved by the Ethical Committee of Animal Welfare and Experimentation of the University of Valencia (Ref#A18348501775). Protocols adhered to Spanish (Real Decreto 53/2013) and European (2010/63/UE) regulations.

### Pharmacological treatment of primary infections

Cure of the primary infections was achieved by pharmacological treatment with PZQ. Mice were treated with a double dose of 100 mg/kg of PZQ at 4 weeks post-primary infection (wppi), orally administered on alternate days [28]. All mice treated with PZQ reverted to negative, as determined by coprological examination. The effect of the pharmacological treatment on the studied parameters was not analyzed since five mice were left uninfected, treated with PZQ as described above and analyzed.

### Treatment of mice with blocking antibodies or isotype-matched IgG control antibodies

Briefly, several mice were sensitized by intraperitoneal injection with specific commercial blocking antibodies, recombinant proteins or isotype-matched control antibodies.

To neutralize the effect of IL-25 produced nonspecifically after the cure of the primary *E. caproni* infection and to investigate the effect of this cytokine in a secondary challenge infection and the role of signal transducer and activator of transcription 6 (STAT6) activation in resistance to infection, we treated two groups of 5 mice each with monoclonal anti-mouse IL-25 (mα-IL-25) (R&D Systems, Minneapolis, MN, USA) or monoclonal anti-mouse IL-4Rα (mα-IL-4Rα) (BioLegend, San Diego, CA, USA). In each group, mice were primarily infected with 50 metacercariae of *E. caproni* and treated with PZQ at 4 wppi. On each of the 2 days prior to a secondary infection at 6 wppi, mice in one group were intraperitoneally injected with mα-IL-25 (concentration 0.25 μg/μl; in 150 μl saline buffer) and mice in the other group were intraperitoneally injected with mα-IL-4Rα (concentration 0.1 μg/μl; in 150 μl saline buffer). Additionally, as control groups, five mice were injected with rat IgG1 (control group for mα-IL-25-treated mice), and five mice were injected with IgG2b (control group for mα-IL-4Rα-treated mice). All mice were sacrificed at 2 weeks post-secondary infection (wpsi).

The effect of recombinant cytokines on the course of the infection was analyzed. For this purpose, groups of five mice were intraperitoneally injected with IL-4 (rIL-4; Prepothech), IL-13 (rIL-13; Prepotech, London, UK) or IL-25 (rIL-25; R&D Systems) (concentration 0.2 μg/μl each) in 150 μl of phosphate buffered saline (PBS) during each of the 4 days after the primary infection with 50 metacercariae of *E. caproni*. All mice were sacrificed at 2 wpsi. Additionally, a group of five mice were treated with recombinant IL-13Rα2 (rIL-13Rα2; R&D Systems) (concentration: 0.2 μg/μl) following the same protocol to study the role of IL-13Rα2 in *E. caproni* infection. As control animals, five mice were intraperitoneally injected with 150 μl of PBS following the same protocol.

### Analysis of IL-25 gene expression over time and its influence in resistance to secondary infections

To analyze further the role of the innately produced IL-25 and the ability of *E. caproni* to induce IL-25 mRNA expression in mice with secondary infections in terms of memory response, we delayed the challenge infection until IL-25 gene expression had recovered to baseline levels. Then, as primary infection, we infected a total of 20 mice with metacercariae of *E. caproni* and treated the infected mice with pzq at 4 wpi. From 6 wppi onwards, three of these mice were sacrificed every 2 weeks, and the levels of IL-25 mRNA expression were studied by real time (RT)-PCR. Once baseline levels were recovered, the remaining five mice of the group were secondarily infected and necropsied at 12 wppt. Moreover, five other mice were used as controls. To avoid age-related changes in susceptibility affecting accurate comparisons, the mice primarily infected were of the same age as those that were secondarily infected.

### Total RNA extraction

Total RNA was extracted from full-thickness sections of ileum of necropsied mice. Total RNA was isolated using the Real Total ARN Spin Plus kit (Durviz SL, Valencia, Spain) according to the manufacturer’s instructions. The cDNA was synthesized using the High Capacity cDNA Reverse Transcription kit (Applied Biosystems, Foster City, CA, USA).

### RT-PCR and relative quantification analysis

For the quantitative PCR, 40 ng total RNA was reverse transcribed to cDNA and added to 10 µl of TaqMan Universal PCR Master Mix, No AmpErase UNG (2×), 1 µL of the specified TaqMan Gene Expression Assay (all Applied Biosystems) and water to a final reaction volume of 20 µL. Reactions were performed on the ABI Prism 7000 thermal cylcer (Applied Biosystems) at the following thermal cycler conditions: initial setup of 10 min at 95 °C; 40 cycles of denaturation of 15 s at 95 °C; and 1 min of annealing and extension at 60 °C each. Samples were amplified in a 96-well plate, and the endogenous control, samples and negative controls were analyzed in triplicate in each plate. All TaqMan Gene Expression primers and probes for inducible nitric oxide synthase (iNOS), cytokines and mucins were designed by Applied Biosystems and provided as Inventoried Assays. Details on the assay are provided in ID details are shown in Additional file 1: Table S1. Each assay contains two unlabeled primers and one 6-FAM dye-labeled primer (TaqMan MGB probe). Primer concentration was optimized by a matrix of reactions testing a range of concentrations for each primer against different concentrations of the partner primer and negative controls were also included.

The cycle threshold (Ct) value was calculated for each sample, housekeeper and uninfected control. The β-actin gene was used as housekeeping gene to normalize for differences in efficiency of sample extraction or cDNA synthesis. To estimate the effect of infection on gene expression levels we used a comparative quantification method (2^−ΔΔCT^ method), which is based on the fact that the difference in threshold cycles (ΔCt) between the gene of interest and the housekeeping gene is proportional to the relative expression level of the gene of interest. The fold change in the target gene was normalized to β-actin and standardized to the expression at time 0 (uninfected animals) to generate a relative quantification of the expression levels.

### Analysis of goblet cell responses

Goblet cell responses to *E. caproni* infections in the ileum of mice were evaluated in primary and secondary infections in rIL-25-treated mice. At each time point, five mice in each group were necropsied, and ileal sections of about 0.7 cm in length were obtained and fixed in 4% paraformaldehyde. After embedding in paraffin wax, serial 4-μm sections were cut from each tissue block and stained with alcian blue. Cell counts were calculated as the number of goblet cells per crypt unit studied over ten selected high-power fields (magnification ×400).

### Indirect immunofluorescence

Translocation and phosphorylation of STAT6 were studied by fluorescent immunohistochemistry on paraffin-embedded tissue sections [29]. The anti-STAT6 (Thermo Fisher Scientific, Waltham, MA, USA) and anti-p-STAT6 (Thermo Fisher Scientific) rabbit antibodies were used. Anti-STAT6 and anti-p-STAT6 were diluted 1/200 and 1/20, respectively, in PBS containing 0.3% Triton X-100 and 10% Flow Cymetry Standard (FCS) and incubated for 2 h in a humid chamber at room temperature, under continuous agitation. After three washes in PBS, intestinal sections were incubated for 2 h with the secondary antibody, goat anti-rabbit IgG conjugated with Alexa Fluor® 647 (Jackson ImmunoResearch Laboratories, Inc., West Grove, PA, USA) and diluted in PBS-Triton™ X-100 (0.3%) (dilution 1/600 for anti-STAT6 and 1/100 for anti-p-STAT6). The slides were washed in PBS, and cell nuclei were counterstained with DAPI before mounting with Fluoromoun (Sigma-Aldrich, St. Louis, MO, USA). Cell staining was analyzed by fluorescence microscopy. The results were studied over five selected high-power fields.

### Enzyme immunohistochemistry

To analyze the tuft cells and cells positive for GATA binding protein 3 (GATA3+ cells), such as type-2 innate lymphoid cells (ILC2s) and Th2 populations in primary and secondary infections with *E. caproni*, enzymatic immunohistochemistry of intestinal sections was performed in a total of 20 mice at 2 wppi (*n* = 5), 2 wppt (*n* = 5) and 2 wpsi (*n* = 5). Additionally, five naïve mice were used as controls. The intestinal samples of these mice were first dewaxed by incubation for 20 min in an oven at 60 °C, then passed through a clearing and dehydration series: four 5-min washes in xylene, then passage through an ethanol series of 100% ethanol [two 3-min washes], 90% ethanol [two 3-min washes] and 70% ethanol [two 3-min washes]) and incubation in 10 mM sodium citrate buffer for 10 min to improve antigen detection. Once the samples were cooled, they were kept in running water for 10 min and washed twice for 5 min in Tris Buffer Saline (TBS) + 0.1% Triton X-100 pH 7.6 with stirring. Sections were blocked with 2.5% Normal Goat Serum (Vector Laboratories, Burlingame, CA, USA) for 1 h at room temperature. After blocking, they were incubated over night at 4 °C with the primary antibody at 1:1000 dilution in TBS and 1% bovine serum albumin (BSA). The primary antibodies were: a-DCLK-1 (Abcam, Cambridge, UK) for labeling tuft cells and a-GATA3 (Abcam) for labeling GATA3+ cells.

In order to eliminate the intrinsic autofluorescence from the tissue, the samples were first incubated in Dual Endogenous Enzime Block (Dako, Agilent Technologies, Santa Clara, CA, USA) for 10 min at room temperature, then with the secondary Polyclonal Goat anti-Rabbit Immunoglobulins/HRP (Dako) antibody diluted 1:1000 in TBS + 0.1% Triton X-100 and 1% BSA for 1 h at room temperature. After all the incubation steps were completed the samples were washed twice for 5 min each time in TBS + 0.1% Triton X-100 with gentle agitation.

DAB (3,3′-diaminobenzidine) was selected as a chromogen to reveal the reaction (Liquid DAB + Substrate Chromogen System; Dako). The development time was controlled by observing the brown precipitates produced by reacting the DAB with the secondary Polyclonal Goat anti-Rabbit Immunoglobulins/HRP antibody. The samples were washed with running tap water to stop the reaction.

Finally, the sections were contrasted with Mayer’s Hematoxilin (Dako), passed through a dehydration chain (Scott’s tap water for 30 s; series of 90% ethanol [two 30-s washes],100% ethanol [two 3-min washes] and xylene [two 3-min washes) and mounted in DPX liquid medium for later analysis under an optical microscope (magnification ×200). Cell populations were studied over ten selected representative high-power fields.

### Statistical analysis

The Chi-squared test (χ^2^) test was used to compare both groups of mice at each week of post-infection. To compare the worm recovery between primary and challenge infections, a Student’s *t* test was used at each week of post-infection. One-way analysis of variance with the Bonferroni test was used as post hoc analysis to compare the gene expression levels of cytokines, enzymes or other genes analyzed by PCR. *p* < 0.05 was considered to indicate statistical significance. Prior to analyses, data were log transformed to achieve normality and verified by the Anderson–Darling test.

## Results

### Treatment of mice with rIL-4 or rIL-13 does not induce resistance to primary infection

The results obtained show that neither treatment with rIL-4 nor treatment with rIL-13 induced resistance to the primary infection. The worm recovery in rIL-4- [range 51–65%; mean ± standard deviation (SD) 61.2 ± 11.2%], rIL-13-treated (62–85%; 76.1 ± 16.5%) or non-treated mice (54–68%; 62.1 ± 10.6%) was very similar.

Treatment of mice with rIL-4 induced decreases in the expression of IL-13 and IL-12p35. Infection of treated mice elicited a marked downregulation of type-1 cytokines, such as IL-12p35, IL-12-p40 or IFN-γ. In rIL-4-treated mice, a significant upregulation of IL-13 was also observed (Additional file 2: Fig. S1a). No significant differences between groups were detected in the remaining cytokines nor in the expression of markers of macrophage activation (data not shown).

Goblet cell counts in naïve mice ranged from 6.1 to 9.4 cells per crypt (mean ± SD: 8.1 ± 1.2 cells per crypt). Treatment with rIL-13 induced significant goblet cell hyperplasia (13.1 ± 2.5 cells per crypt) that was more pronounced after infection of the treated mice (19.3 ± 4.3 cells per crypt). In contrast, hyperplasia after the experimental infection only was observed in the animals treated with rIL-4 (15.3 ± 2.9 cells per crypt). RELM-β expression was downregulated in animals treated with rIL-4 but, in contrast, the values were greater in the controls in rIL-13-treated mice at 2 wppi (Additional file 2: Fig. S1b).

### Secondary *E. caproni* infection induces expansion of tuft cells and GATA3+ cells concomitantly with a Th2 response

Primary infection did not elicit hyperplasia of tuft cells and only elicited a slight increase in GATA3+ cells populations, although this increase rapidly returned to basal values. No increase was observed after treatment with PZQ in either cell type. In contrast, a marked increase in counts of tuft cells and GATA3+ cells was observed as a consequence of the secondary infection (Additional files 3, 4: Figs. S2 and S3).

### Blocking of IL-25 reverts resistance against *E. caproni* challenge infections

To analyze the effect of IL-25 in resistance to *E. caproni* challenge infections, a total of ten mice were given a primary infection and treated with PZQ at 4 wpi. Five of these mice were treated with mα-IL-25 before a challenge *E. caproni* infection at 2 wppt; the remaining five mice were given a secondary infection at the same time without mα-IL-25 treatment. All mice were necropsied at 2 wpsi.

**Fig. 1:**
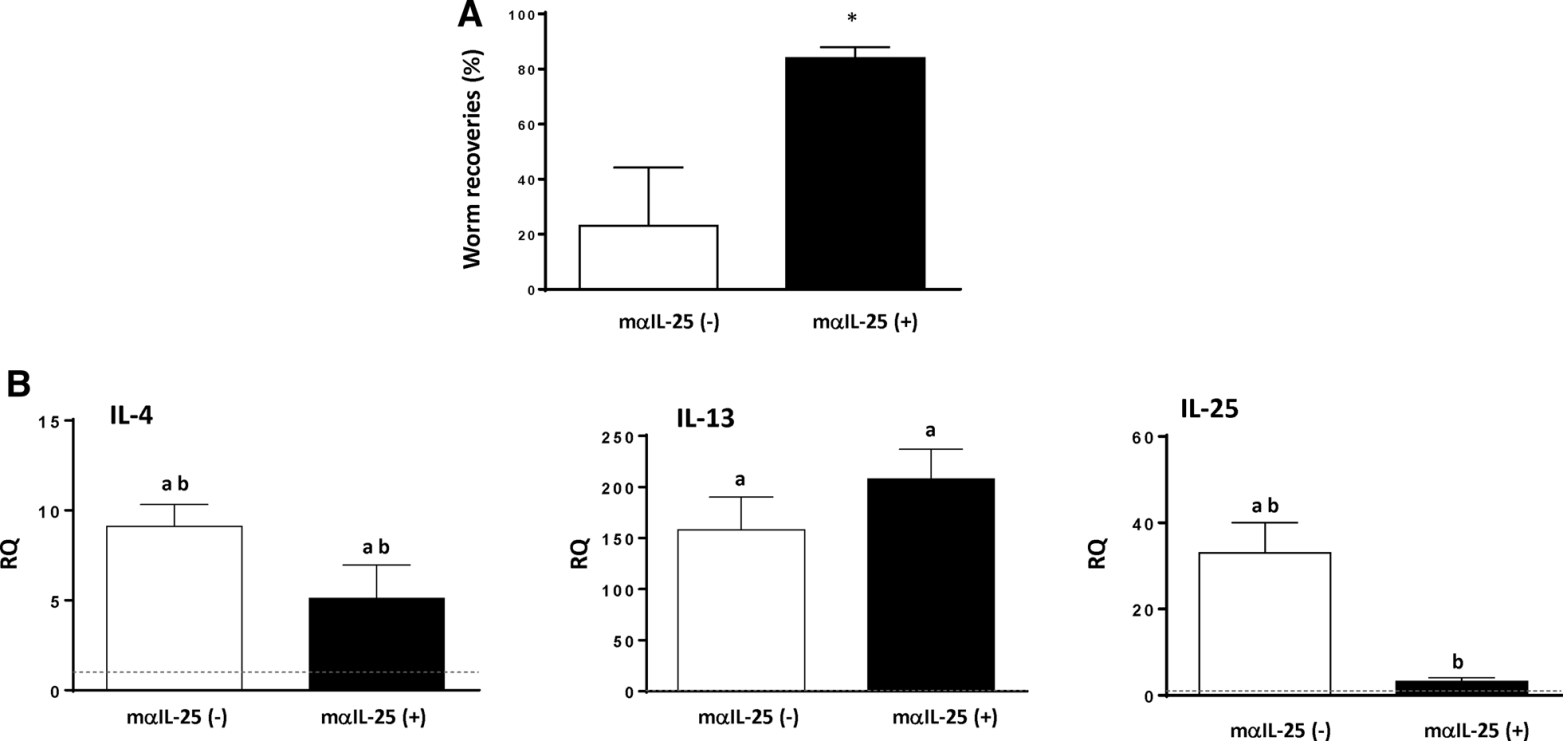
Blocking of interleukin-25 (*IL-25*) in challenge infections with *Echinostoma caproni* reverts resistance to infection despite the development of a T-helper cell (Th2) response. **A** Worm recovery in IL-25 monoclonal antibody (*mα-IL-25*)-treated ICR mice and non-treated mice after a challenge infection with *E. caproni*. **B** Expression of cytokine mRNA in the intestinal tissue of mα-IL-25-treated ICR mice and non-treated mice after a challenge infection with *E. caproni*. The relative quantities (*RQ*) of cytokine genes are shown after normalization with β-actin and standardization of the relative amount against the day 0 sample. Vertical bars represent the standard deviation (SD).** A **Asterisk indicates significant difference from non-mα-IL-25-treated mice,** B** lowercase letters (*a*, *b*) above bars indicate either significant differences with respect to naïve mice controls (*a*) or significant differences between groups (*b*), both at *p* < 0.05

The results obtained show that blockade of the IL-25 reverted the partial resistance to infection and that the number of worms recovered was significantly higher in the animals treated with mα-IL-25 than in the non-treated mice at necropsy (Fig. 1a). Worm recoveries in mα-IL-25-treated mice ranged from 78–89% (mean ± SD: 84.00 ± 3.9), whereas the values ranged from 9 to 32% (23.12 ± 8.2) in non-treated animals.

Changes in cytokine expression in secondary infection at 2 wpsi in relation to the blockade of IL-25 were investigated by RT-PCR. The most relevant alterations affected IL-4, IL-13 and endogenous IL-25. Blockade of IL-25 resulted in a significant overexpression of IL-4 and IL-13 and a reduction in the expression of endogenous IL-25 (Fig. 1b).

Animals treated with mα-IL-25 showed a similar cytokine profile to non-treated mice, and in both groups, a type-2 response was generated with elevated gene expression of IL-4 and IL-13 after the challenge infection. The most striking feature observed is likely the significantly lower gene expression of endogenous IL-25 in mα-IL-25-treated animals being similar to naïve levels (Fig. 1b).

**Fig. 2:**
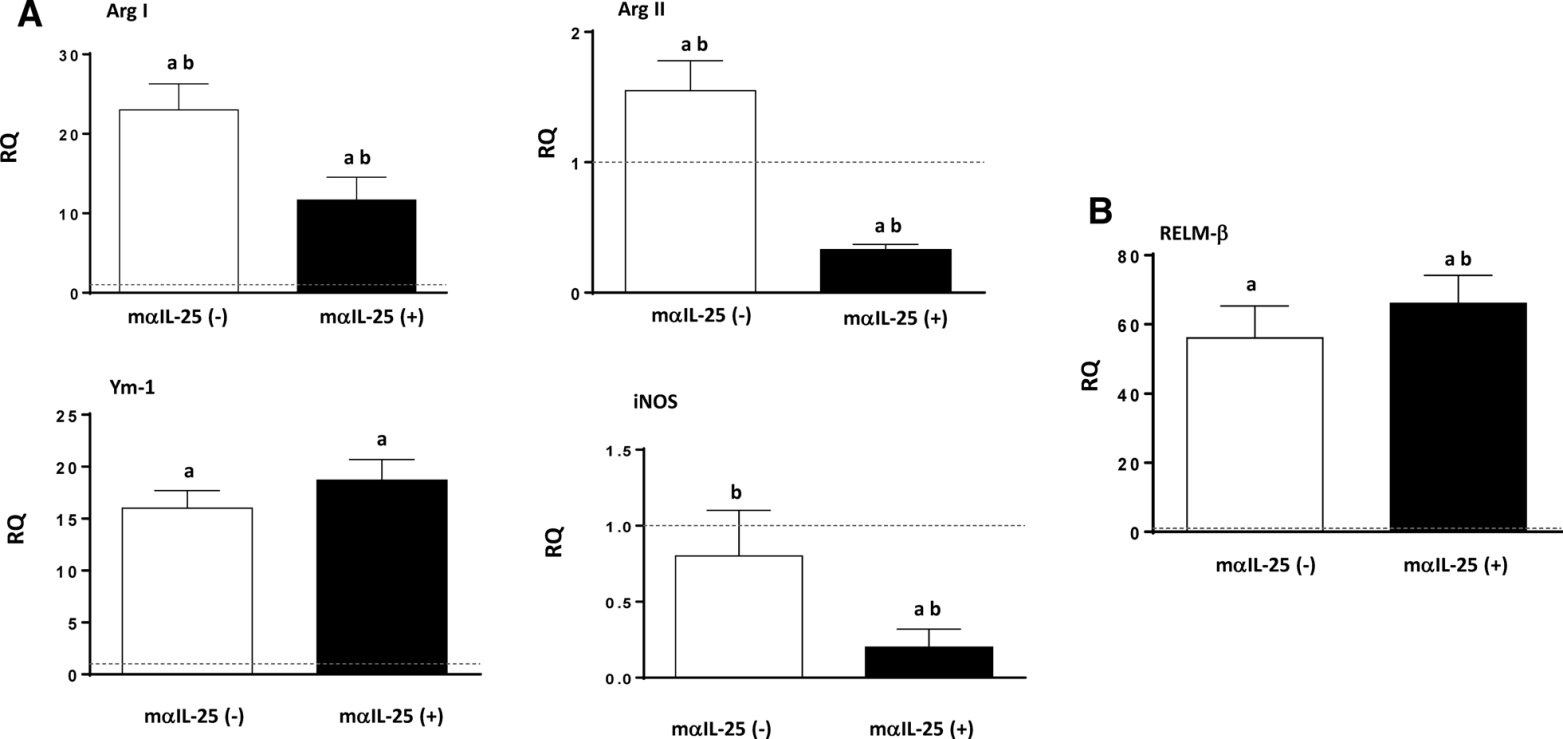
Blocking of IL-25 induced alternative activation of macrophages after challenge infection. **A** Pattern of macrophage activation is different in primary and secondary infections analyzed according to the gene expression of marker mRNA of both classical (ArgII and iNOS) and alternative (ArgI and Ym-1) macrophage activation in the intestinal tissue of mα-IL-25-treated mice and non-treated mice after a challenge infection with *E. caproni***. B** Expression of resistin-like molecule beta (*RELM-β*) mRNA in the intestinal tissue of mα-IL-25-treated ICR mice and non-treated mice after a challenge infection with *E. caproni*. The RQ of cytokine genes are shown after normalization with β-actin and standardization of the relative amount against the day 0 sample. Vertical bars represent the SD. Lowercase letters (*a*, *b*) above bars indicate either significant differences with respect to naïve mice controls (*a*) or significant differences between groups (*b*), at *p  *< 0.05

To study the changes in macrophage activation induced by the blockade of IL-25, we analyzed markers of classical macrophage activation (M1; ArgII and iNOS) and alternative activation (M2; Ym-1 and ArgI). Interestingly, antibody blockade of endogenous IL-25 did not change the predominance of M2 activation, despite a slight decrease in ArgI gene expression. However, a significant decrease in the expression of ArgI, ArgII and iNOS was observed with respect to the group of non-treated mice (Fig. 2a). Moreover, blocking of IL-25 did not induce changes in RELM-β expression (Fig. 2b).

### Return to baseline expression of IL-25 after healing of the primary infection ablated the resistance against challenge infection

**Fig. 3:**
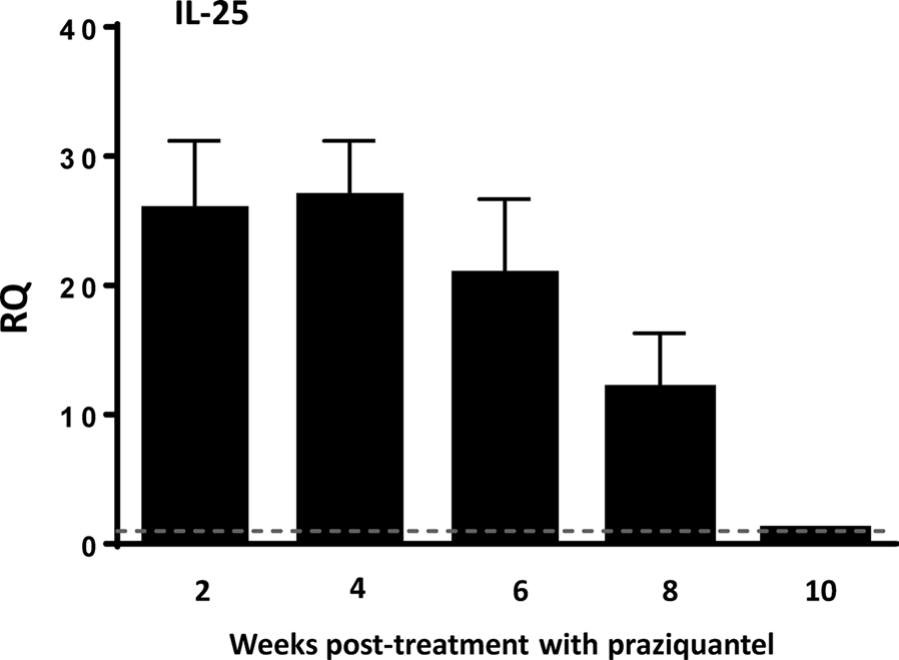
Interleukin-25 (*IL-25*) gene expression returned to baseline levels 10 weeks after pharmacological cure of a primary infection. Expression of IL-25 mRNA in the intestinal tissue mice after curation with praziquantel of a primary infection with *E. caproni*. The RQ of cytokine genes are shown after normalization with β-actin and standardization of the relative amount against the day 0 sample. Vertical bars represent the SD

To analyze further the role of the innately produced IL-25 and the ability of *E. caproni* to induce IL-25 expression in mice in memory secondary infections, we delayed the challenge infection until IL-25 gene expression had recovered to baseline levels and compared it with other animals of the same age but primarily infected. Baseline levels of IL-25 mRNA in treated mice were recovered at 10 wppt with values statistically similar to those of the naïve controls (Fig. 3).

**Fig. 4:**
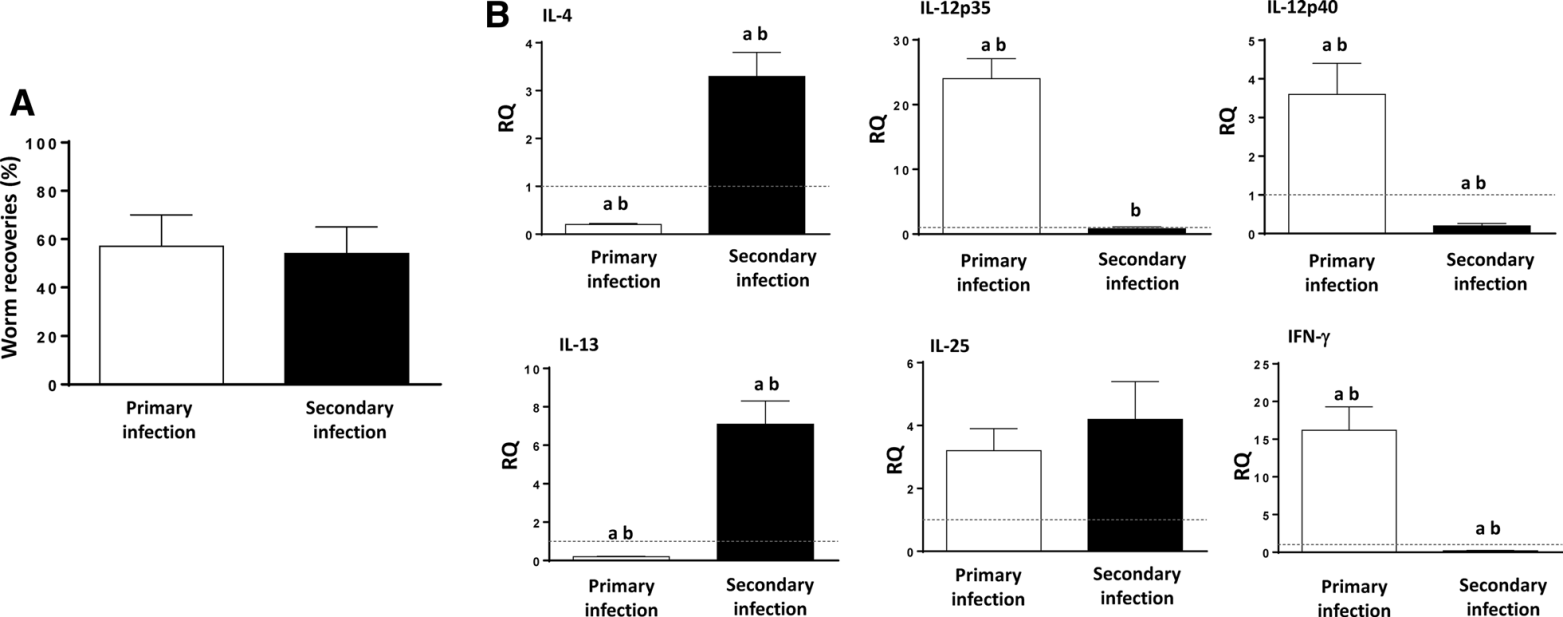
Recovery of baseline expression of IL-25 mRNA after healing of the primary infection reverted the resistance against challenge infection together with a Th2 response. **A** Worm recovery of a primary infection in naïve mice and in that of a challenge infection in mice in which the basal levels of mRNA expression were recovered after the cure of a primary infection. **B** Expression of cytokine mRNA in the intestinal tissue of both groups of mice at 2 weeks after the primary and secondary infection (wppi and wpsi, respectively). The RQ of cytokine genes are shown after normalization with β-actin and standardization of the relative amount against the day 0 sample. Vertical bars represent the SD. Lowercase letters (*a*, *b*) above bars indicate either significant differences with respect to naïve mice controls (*a*) or significant differences between groups (*b*), at *p  *< 0.05

Results of worm recovery show that after the levels of IL-25 mRNA expression had declined, mice were once again susceptible to infection. The worm recovery in secondary infections in the absence of IL-25 (46–59%; mean ± SD: 54.2 ± 11.1%) was similar to that of observed in the animals of the same age primarily infected [49–61%; 58.6 ± 13.6) (Fig. 4a).

The profile of cytokine mRNA expression showed that secondary infection at 10 wppt induced the development of a Th2 phenotype despite the lack of IL-25 at the time of infection (Fig. 4b). Secondary infection at 10 wppt was characterized by the significant upregulation of IL-4 and IL-13 and unaltered or downregulation of type-1 cytokines, such as, IL-12p35/IL-12p40 or IFN-γ. In contrast, the primary infection induced a Th1 response. No differences between groups were observed in relation to endogenous IL-25 expression (Fig. 4b).

### Activation of STAT6 is not required for resistance against *E. caproni*

To evaluate the role of STAT6 activation in the resistance mediated by IL-25, we used two experimental approaches in resistant rIL-25-treated mice. In one group of mice IL-4Rα was blocked using monoclonal antibodies prior to a primary infection with 50 metacercariae; a second group of mice was treated with rIL-13Rα2 prior to the infection; a third group of rIL-25-treated mice were exclusively infected with 50 *E. caproni* metacercariae and used as control of the effect of treatment with IL-4Rα and/or rIL-13Rα2. All mice were necropsied at 2 wppi.

All 15 mice were refractory to infection in relation to the treatment with rIL-25. In two of the groups of animals, one with blocked IL-4Rα and one with rIL-13Rα2 treatment prior to infection, a significant reduction in STAT6 phosphorylation and translocation was observed at 2 wppi compared with the to rIL-25-treated control animals. In the group of animals treated with anti-IL-4Rα, the signal was similar to that observed in naïve animals (Additional file 5: Fig. S4).

**Fig. 5:**
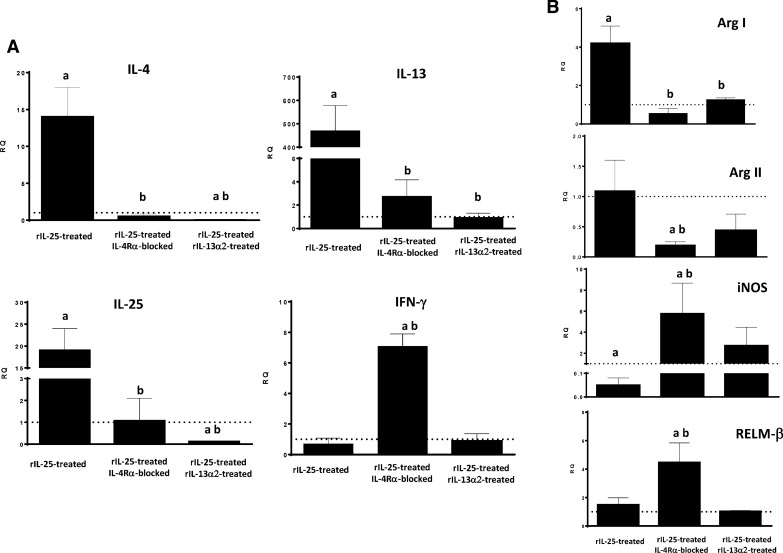
Treatment of mice with either mα-IL-4Rα or rIL-13Rα2 abrogates Th2 response and alters the pattern of macrophage activation despite the presence of IL-25. **A** Expression of cytokine mRNA in the intestinal tissue IL-25-treated-mice that were also treated with monoclonal anti-mouse IL-4Rα* (mα-IL-4Rα*) or recombinant IL-13Rα2 (*rIL-13Rα2*) at 2 weeks post-primary infection (wppi) with *E. caproni*; **B** Pattern of macrophage activation analyzed by the expression of marker mRNA of both classical (ArgII and iNOS) and alternative (ArgI and Ym-1) macrophage activation in the intestinal tissue of IL-25-treated-mice that were also treated with mα-IL-4Rα or rIL-13Rα2 at 2 wppi with *E. caproni*. The RQ of cytokine genes are shown after normalization with β-actin and standardization of the relative amount against the day 0 sample. Vertical bars represent the SD. Lowercase letters (*a*, *b*) above bars indicate either significant differences with respect to naïve mice controls (*a*) or significant differences between groups (*b*), at *p  *< 0.05

However, the response generated after exposure to metacercariae was different in these groups of mice. A type-1 phenotype developed with elevated levels of IFN-γ gene expression and no significant changes in IL-4 and IL-13 with respect to naïve controls. No significant changes as compared with naïve mice were observed either in the endogenous expression of IL-25 mRNA or in the expression of the other cytokines studied. In contrast, treatment of mice with rIL-13Rα2 abrogated any response to infection and no increase in the expression of any cytokine was observed. A decline in the expression of IL-4 and endogenous IL-25 with respect to naïve mice was observed (Fig. 5a). The expression of the remaining cytokines remained unaltered in this group of mice. Moreover, slight increases in the expression of iNOS and RELM-β were observed in the group of mice treated with anti-IL-4Rα (Fig. 5b).

**Fig. 6:**
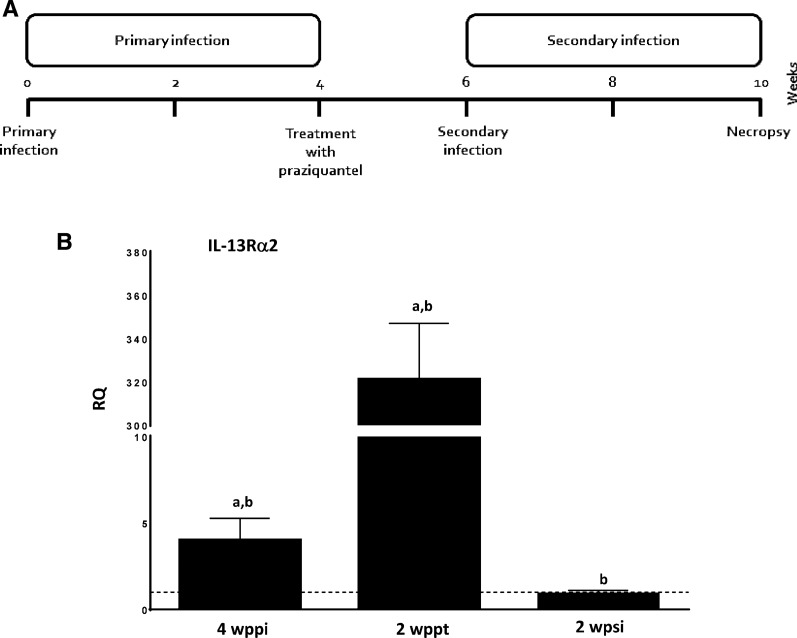
Pharmacological curation of an *E. caproni* primary infection exacerbates the expression of IL-13Ra2. **A** Schematic representation of the experimental protocol. **B** Levels of mRNA expression of IL-13Ra2 in the intestinal tissue of mice primarily infected at 4 wpi, at 2 weeks post-treatment with praziquantel and at 2 wpsi. The relative quantities (RQ) of cytokine genes are shown after normalization with β-actin and standardization of the relative amount against day 0 sample. Vertical bars represent the standard deviation. Lowercase letters (*a*, *b*) above bars indicate either significant differences with respect to naïve mice controls (*a*) or significant differences between groups (*b*), at *p  *< 0.05

The expression of IL-13Rα2 in different situations was studied by quantitative PCR. Primary infection resulted in a slight increase in the expression, which increased greatly after PZQ treatment and just before secondary infection associated with resistance. Expression declined to negative values after secondary infections (Fig. 6).

## Discussion

The results show that primary infection of mice with *E. caproni* did not elicit IL-25 upregulation, but pharmacological cure of the primary infection induced a marked overexpression of IL-25. Due to these elevated levels of IL-25 gene expression, mice became resistant to a challenge infection at 2 wppt, concomitantly with the development of a robust Th2 response [26, 27]. This study extends our previous investigations, being designed to analyze the role of IL-25 in the generation resistance to *E. caproni* infections in mice. The study focused on factors determining IL-25 upregulation, the immune regulatory role of IL-25 and the effector mechanisms induced by IL-25 that determine resistance.

Although it is generally accepted that IL-25 is critical for the development of resistance against intestinal helminths, there is no consensus regarding the mechanisms by which this cytokine enhances resistance. Traditionally, the role of IL-25 in resistance has been exclusively attributed to its immune regulatory activity. IL-25 promotes Th2 immunity through the production of IL-4 and/or IL-13 which, in turn, induce STAT6-mediated intestinal alterations determining parasite rejection [9, 30–33]. Upon helminth establishment, intestinal tuft cell populations expand and release IL-25 that in turn activates a variety of immune cells to initiate type-2 responses and promote Th2-cell-mediated immunity. In response to IL-25 and other alarmins, ILC2s produce large amounts of IL-13, polarizing naïve CD4+ T cells into Th2. Antigen-presenting cells, such as basophils and dendritic cells, are also activated and induce Th2 polarization through different mechanisms [6, 13, 15]. This mechanism is consistent with our results, since we observed that in resistant secondary infections against *E. caproni* there were an expansion of the populations of tuft cells and GATA3+ cells. The effector mechanisms activated in the resistance response against *E. caproni* seem to be dependent on the expansion of the tuft cells, thereby promoting the overexpression of IL-25 and activation of ILC2s or Th2 cells. In an environment of helminth infection, IL-25 acts as a mediator of the activation of ILC2s promoting the polarization of the immune response towards a Th2 phenotype [34].

A number of laboratories have reported that IL-25 upregulation is induced by infection with intestinal helminths, such as *Nippostrongylus brasiliensis*,* Trichinella spiralis*,* Trichuris murisor* and *Heligmosomoides polygyrus*, leading to activation of type-2 responses and resistance to infection [9, 30–33, 35]. Despite these studies, recent works have suggested that the regulatory role of IL-25 may be secondary and that this cytokine operates autonomously from Th2 response in the generation of resistance against intestinal helminths [8, 16]. Smith and co-workers [8] showed that IL-25 plays a more important role than simply the promotion of protective Th2 responses. In fact, these authors demonstrated that adaptive Th2 response to *H. polygyrus* in mice developed normally even in the absence of IL-25R activation, but that effector mechanisms became impaired. Similarly, Mearns et al. [16] challenged the role of IL-25 in the promotion of Th2 responses in intestinal helminth infections. Using crossed IL-25^−/−^ C57BL/6 mice and 64 IL-4 C57BL/6 reporter mice, these authors demonstrated no physiological role for IL-25 in either the differentiation of Th2 cells or their development to effector or memory Th2-cell subsets. For example, IL-25 deficient mice mounted normal Th2 responses following *N. brasiliensis* infections. Our results indicate that the involvement of IL-25 in intestinal helminth infections may be more complex than previously expected. IL-25 is required for resistance against *E. caproni* infection in mice, but this resistance is independent of IL-4 and/or IL-13 activity and STAT6 activation. Furthermore, IL-25 may have a role in promoting Th2 responses although its contribution is different in primary and memory secondary responses. IL-25 is required for the development of a Th2 phenotype in response to primary infections but, in contrast, memory response is characterized by the upregulation of type-2 cytokines despite the lack of IL-25, suggesting that IL-25 enhances the expansion memory cells.

The inability of mice to produce IL-25 in response to primary infection and, consequently, IL-13 results in susceptibility to infection. However, treatment of mice with rIL-25 induces resistance to infection, concomitantly with elevated levels of IL-13 [26]. Our results confirm that the development of a Th2 response relies on the presence of IL-13 and STAT6 activation. Despite the lack of IL-25, in our study, treatment of mice with rIL-4 or rIL-13 elicited both a Th2 phenotype in response to *E. caproni* primary infection and the activation of several IL-13-mediated mechanisms, such as goblet cell hyperplasia or RELM-β activation. Our results also support the notion that STAT6 activation is required for the production of type-2 cytokines in response to *E. caproni* primary infection. Blocking of IL-4Rα in mice treated with rIL-25 induced a decline in STAT6 phosphorylation and a Th1 response to infection. Furthermore, our results suggest that IL-13Rα2 plays an important role in the regulation of the response to primary infection. Treatment of mice with IL-13Rα2 abrogated the immune response to *E. caproni* primary infection despite the presence of rIL-25.

IL-4 and IL-13 share a common receptor, namely the IL-4Rα chain, but IL-13 also uses IL-13Rα1 for signaling via JAK1 and JAK2 of the Janus kinase (JAK) family of tyrosine kinases. IL-13 binds 13Rα1 which complexes with IL-4Rα to form the type-1 signal receptor, but IL-13 also binds the cell surface and soluble forms of the monomeric type-2 receptor (IL-13Rα2 chain). However, IL-13Rα2 has a decoy effect, lacking signal transduction machinery and limiting the activity of IL-13 since it binds the cytokine and makes it unavailable for activating the type-1 receptor [36–39]. Herein, we have shown that the rIL-13Rα2 chain limits the ability of mice to respond to *E. caproni* primary infection, even in the presence of rIL-25. Treatment of mice with both rIL-25 and rIL-13Rα2 abrogated the response to *E. caproni* infection, and no changes in cytokine gene expression were observed as a consequence of the infection. IL-13Rα2 may act as negative regulator of both IL-13, inhibiting signal transduction, and STAT6 activation through the preferential binding of IL-13 to IL-13Rα2 [40]. However, IL-13Rα2 also inhibits IL-4-induced STAT6 activation and interacts with IL-4Rα, even in the absence of IL-13. IL-13Rα2 probably blocks the activation of STAT6 by physical interaction between the short domain and the cytoplasmic domain of the IL-4Rα chain that harbors the STAT6 docking sites [36, 39, 40]. In fact, we have observed that IL-13Rα2 expression when highly upregulated coincides with the resistance to secondary infection at 2 wppt.

In contrast to the situation that occurs in primary infections, IL-25 does not appear to be required for the development of Th2 responses in secondary *E. caproni* infections. Our study shows that the mice were also unable to produce IL-25 in a secondary challenge infection, but that even in the absence of IL-25 secondary *E. caproni* infection at 2 wppt induced a Th2 response. This Th2 response was attributed to the presence of elevated levels of IL-25 produced after the cure of the primary infection [26]. However, blocking of the innately produced IL-25 after healing of the primary infection gave rise to a type-2 response as a consequence of the secondary infection, showing that IL-25 was not related to biasing of the immune response. This is in contrast with the results obtained with other intestinal helminths. In *H. polygyrus* infections, both primary and secondary infections included IL-25, but both responses were different. IL-25 response in secondary infections was higher, concomitantly with a more potent Th2 response and enhanced resistance to infection. In contrast, the lower levels of IL-25 overexpression to primary infections was reflected in a weak response of Th2 cytokines and chronic infections [35]. Although IL-25 does not appear to determine the polarization of Th2 cells in secondary *E. caproni* infections, this cytokine could facilitate the development to memory Th2 cell subsets. Mearns and co-workers [16] reported that there was no requirement for IL-25 in the development of Th2 cells during *H. polygyrus* infections. To analyze the role of IL-25 in the generation of memory responses against resistance to *E. caproni*, we delayed the challenge infection until the levels of innate IL-25 gene expression upregulation declined to baseline, which occurred at 10 wppt. Mice were susceptible to the challenge infection despite the development of a Th2 phenotype with elevated levels of IL-4 and IL-13 gene expression but low levels of endogenous expression of IL-25. This result suggests that innately produced IL-25 after the healing of a primary infection is involved in the differentiation of memory cells.

Resistance to *E. caproni* primary infection is associated with IL-4-independent mechanisms and based on IL-13 activity and STAT6 activation [24, 25]. However, recent studies have suggested that mechanisms of resistance to intestinal helminth infections mediated by IL-25 are not dependent on IL-4 and/or IL-13 activity [8]. Our results support the notion that IL-25 operates autonomously from type-2 cytokines and that the generation of resistance is exclusively mediated by IL-25. Treatment of mice with rIL-4 or r IL-13 did not provide resistance to a primary *E. caproni* infection in relation to the lack of IL-25, despite the development of a Th2 response.

Blocking of the IL-13 receptors induced a significant reduction of STAT6 phosphorylation concomitantly with a reduction of goblet cell hyperplasia and downregulation of RELM-β. However, both control animals and those with the blocked IL-13 receptors were refractory to primary infection due to the presence of exogenous IL-25. These results indicate that resistance is exclusively mediated by IL-25—independently of the presence of IL-13 and STAT6 activation, probably in relation to M2 activation. Blocking of the IL-13 receptors took the values of iNOS expression to almost zero, together with overexpression of Arg1, indicating an increased M2 activation. It is well known that IL-25 induces alternative activation of macrophages and that this alternative activation this an important mechanism for parasite rejection [8, 41]. Independently of the presence of IL-13, M2 has been shown to be crucial for immunity against several intestinal helminths, such as *H. polygyrus* [42]. An interesting feature is the upregulation of IL-13 after blocking of its receptors. Smith et al. [8] obtained similar results in rIL-25-treated mice in *H. polygyrus* infections, reporting that M2 may represent a major source of IL-13; these authors also demonstrated that IL-4, in addition with IL-4Rα signaling, is required for M2 activation and parasite elimination. Expression of IL-25R in M2 could be required for the parasite expulsion in the presence of IL-4Rα signaling [8]. Our results support that M2 activation and the subsequent IL-13 overexpression do not depend on type-I receptor signaling. Although the IL-4 expression was not very high, this cytokine may well act via type-I receptor signaling, thereby enhancing the resistance to *E. caproni*. Strikingly, treatment of mice with rIL-4 did not yield either resistance or M2 activation, probably in relation to the lack of IL-25 production. This observation suggests that IL-4, but not IL-13, might be necessary for resistance along with IL-25.

A striking feature of *E. caproni* infections in mice is that tuft cell hyperplasia and the subsequent IL-25 overexpression exclusively occur as a consequence of the healing of the infection. Howitt et al. [14] suggested that IL-25 upregulation in intestinal helminth infection is initiated during colonization through the recognition of parasite compounds by tuft cells via taste chemosensory pathways. For this purpose, tuft cells possess multiple taste-chemosensory G protein-coupled receptors, many of which require the G protein subunit gustducin and the transient receptor potential cation channel subfamily M member 5 (TRMP5) to transduce the signals [43]. Howitt et al. [14] reported that disruption of chemosensory signaling by the loss of TRMP5 abrogated tuft cell expansion and IL-25 upregulation on mice infected with *N. brasiliensis*,* T. spiralis* or *H. polygyrus*. Although other mechanisms of immune suppression cannot be discarded [44], the lack of IL-25 expression in both *E. caproni* primary and challenge infections suggests that parasite components do not activate taste chemosensory pathways in tuft cells of mice, which in turn explains the susceptibility to both type of infections. The fact that expansion of GATA3+ cells exclusively occurs after a secondary infection in the presence of IL-25 may indicate that only the simultaneous combination of signals provided by the parasite and IL-25 are able to induce the polarization to Th2.

## Conclusions

Susceptibility of mice relies in the inability of mice to produce IL-25 in response to infection. In contrast to primary infection, secondary infection elicits a type-2 response, even in the absence of IL-25 expression. Despite the development of a type-2 response, mice are susceptible to secondary infection in relation to the lack of IL-25. Resistance to infection is due to IL-25, which acts autonomously from the Th2 response in the parasite clearance. These results may be of importance for improving our understanding of the mechanisms inducing resistance to infections both in humans and animals.

## Supplementary information

**Additional file 1: Table S1.** Applied Biosystems Inventoried assays used in the present work.

**Additional file 2: Fig. S1.** Treatment of mice with rIL-4 or rIL-13 induces a Th2 response and RELM-β overexpression in response to primary *Echinostoma caproni* and infection but not resistance to infection. (A) expression of cytokine mRNA in the intestinal tissue of rIL-4-treated or rIL-13-treated mice at 2 weeks post-primary infection with *E. caproni*; (B) expression of RELM-β mRNA in the intestinal tissue (F) of naïve mice, non-infected rIL-4- or rIL-13-treated mice and infected rIL-4- or rIL-13-treated mice at 2 weeks post-primary infection. The relative quantities (RQ) of cytokine genes are shown after normalization with β-actin and standardization of the relative amount against day 0 sample. Vertical bars represent the standard deviation. a: significant differences with respect to naïve mice controls; b: significant differences between groups (*p* < 0.05).

**Additional file 3: Fig. S2.** Immunohistochemical images showing changes in tuft cell populations (A) and GATA3+ cells (B) 2 weeks after primary infection (2 wppi), 2 weeks after treatment with praziquantel (2 wppt) and 2 weeks after secondary infection with *E. caproni* (2 wpsi). Scale bar: 10 μm.

**Additional file 4: Fig. S3.** Secondary *E. caproni* infection induces expansion of tuft cells and GATA3 + cells. (A) Schematic representation of the experimental protocol; (B) Counts of tuft cell populations and (C) GATA3 + cells 2 weeks after primary infection (2 wppi), 2 weeks after treatment with pzq (2 wppt) and 2 weeks after secondary infection with *E. caproni* (2 wpsi). Vertical bars represent the standard deviation. a: significant differences with respect to naïve mice controls; b: significant differences between groups (*p* < 0.05).

**Additional file 5: Fig. S4.** Treatment of IL-25-treated-mice with mα-IL-4Rα or rIL-13Rα2 reduces STAT6 activation. Indirect immunofluorescence with anti-STAT6 (red) and anti-STAT6P (red) on intestinal tissue of IL-25-treated-mice that were also treated with mα-IL-4Rα or rIL-13Rα2 at 2 weeks post-primary infection. Scale bar: 30 μm.

## Data Availability

Not applicable.

## Abbreviations

Ct: Cycle threshold
DCLK1: Doublecortin like kinase 1
GATA3: GATA binding protein 3
iNOS: Inducible nitric oxide synthase
JAK: Janus kinase
PZQ: Praziquantel
RELM-β: Resistin-like molecule beta
STAT6: Signal transducer and activator of transcription 6
TRMP5: Transient receptor potential cation channel subfamily M member 5
TSLP: Thymic stromal lymphopoietin
wpi: Weeks post-infection
wppi: Weeks post-primary infection
wppt: Weeks post-treatment with praziquantel
wpsi: Weeks post-secondary infection
