# Supplementary material for: Interleukin-25-mediated resistance against intestinal trematodes does not depend on the generation of Th2 responses
Source: Parasit Vectors. 2020 Dec 4;13:608. doi: 10.1186/s13071-020-04467-7 (PMC7716497; doi:10.1186/s13071-020-04467-7)
Supplement: Supplementary file 1 — Additional file 1: Table S1. Applied Biosystems Inventoried assays used in the present work. [file 13071_2020_4467_MOESM1_ESM.docx]

**Additional file 1: Table S1.** Applied Biosystems Inventoried

assays used

|  | Assay ID Details |
| --- | --- |
|  | Mice |
| β-actin | Mm01205647_g1 |
| IL-2 | Mm00434256_m1 |
| IL-4 | Mm00445259_m1 |
| IL-12p35 | Mn00434165_m1 |

IL-12p40 Mn00434174_m1

| IL-13 | Mm99999190_m1 |
| --- | --- |
| IL-25 | Mm00499822_m1 |
| IFN-γ | Mm99999071_m1 |
| IL-13Rα2 | Mm00515166_m1 |
| Arg I | Mm00475988_m1 |
| Arg II | Mm00477592_m1 |
| Ym-I | Mm00657889_mH |
| iNOS | Mm01309897_m1 |
| RELMβ | Mm00445845_m1 |
