# Supplementary figures and images for: Interleukin-25-mediated resistance against intestinal trematodes does not depend on the generation of Th2 responses
Source: Parasit Vectors. 2020 Dec 4;13:608. doi: 10.1186/s13071-020-04467-7 (PMC7716497; doi:10.1186/s13071-020-04467-7)

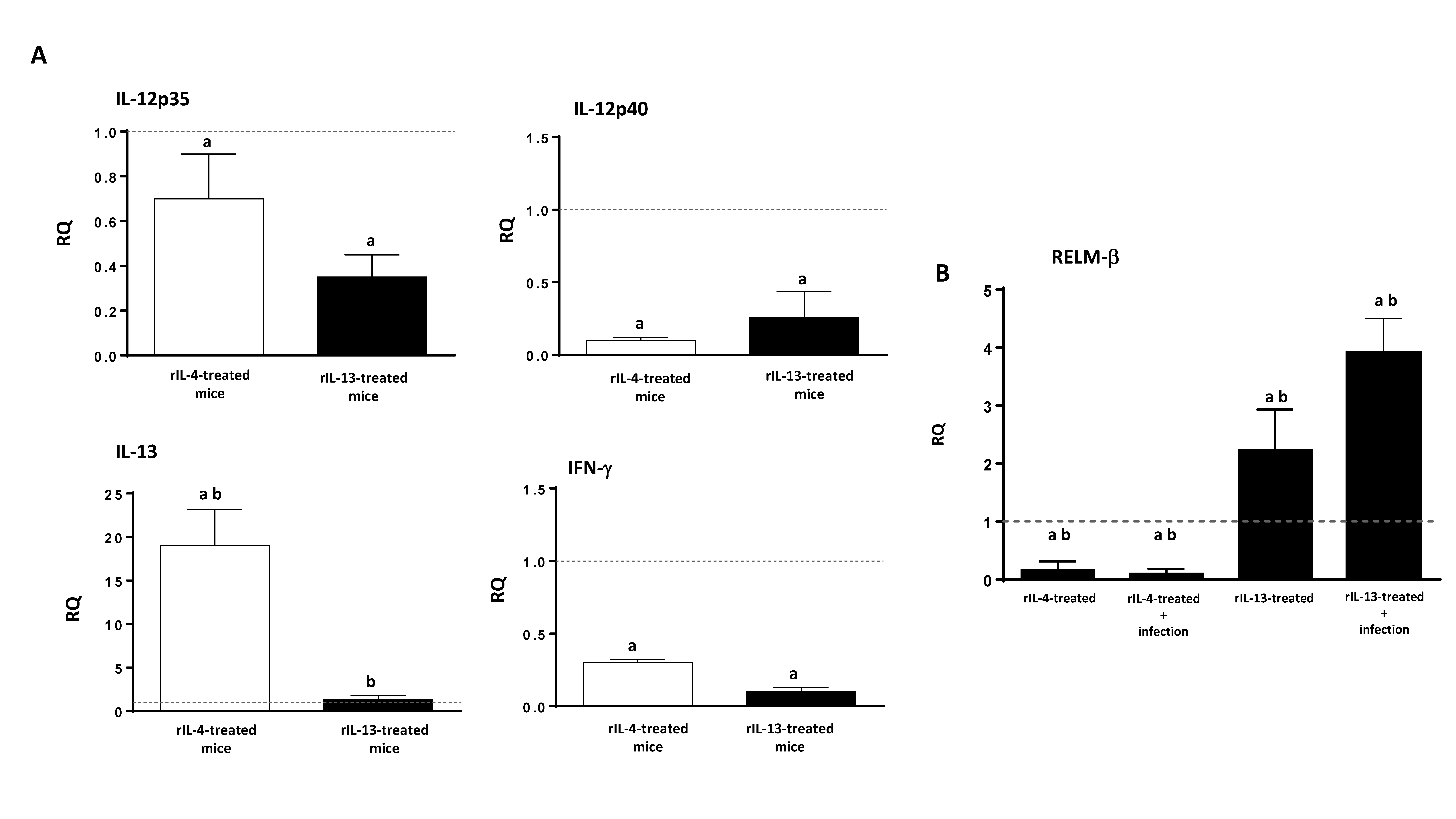

Supplement: Supplementary file 2 — Additional file 2: Fig. S1. Treatment of mice with rIL-4 or rIL-13 induces a Th2 response and RELM-β overexpression in response to primary Echinostoma caproni and infection but not resistance to infection. (A) expression of cytokine mRNA in the intestinal tissue of rIL-4-treated or rIL-13-treated mice at 2 weeks post-primary infection with E. caproni; (B) expression of RELM-β mRNA in the intestinal tissue (F) of naïve mice, non-infected rIL-4- or rIL-13-treated mice and infected rIL-4- or rIL-13-treated mice at 2 weeks post-primary infection. The relative quantities (RQ) of cytokine genes are shown after normalization with β-actin and standardization of the relative amount against day 0 sample. Vertical bars represent the standard deviation. a: significant differences with respect to naïve mice controls; b: significant differences between groups (p < 0.05). [file 13071_2020_4467_MOESM2_ESM.tif]

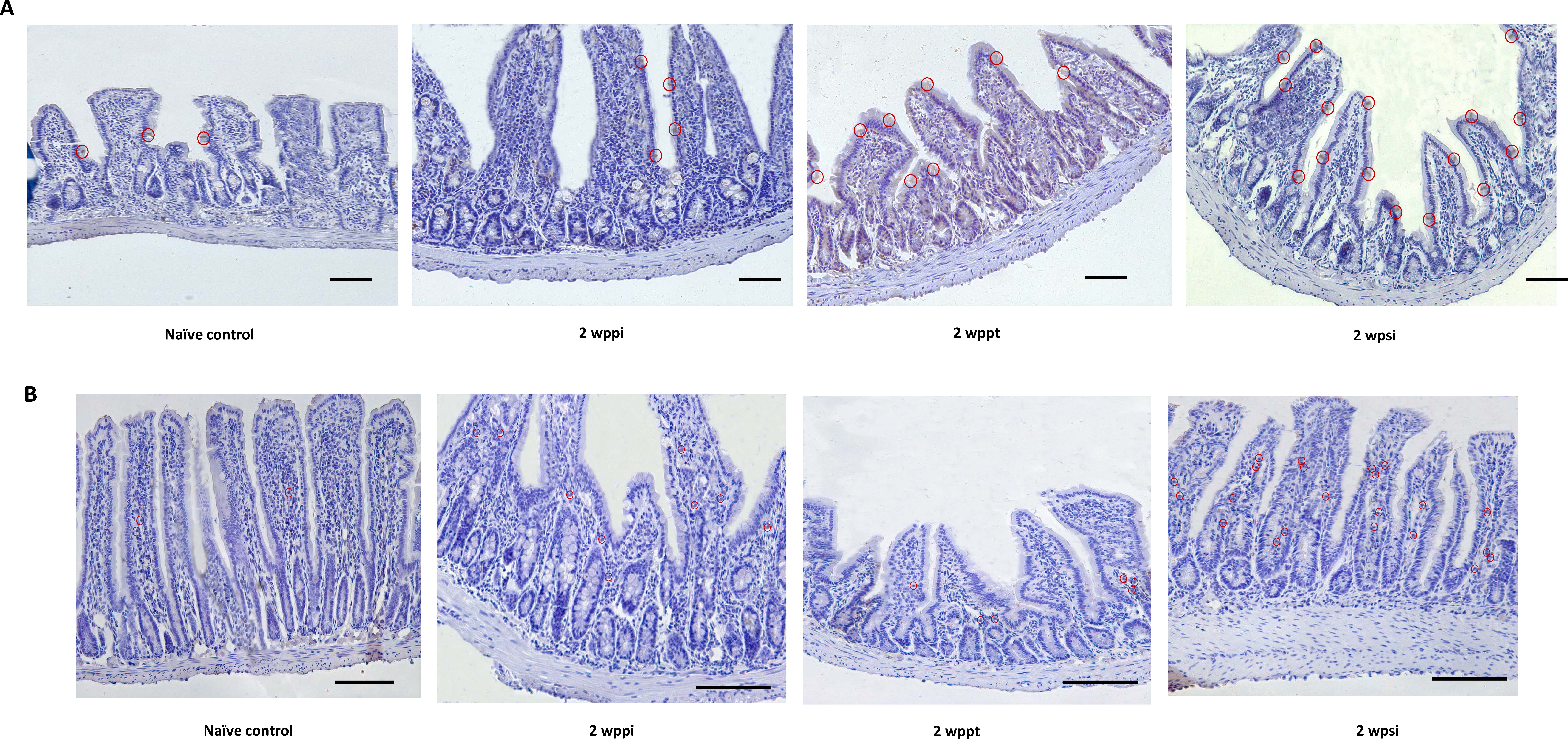

Supplement: Supplementary file 3 — Additional file 3: Fig. S2. Immunohistochemical images showing changes in tuft cell populations (A) and GATA3+ cells (B) 2 weeks after primary infection (2 wppi), 2 weeks after treatment with praziquantel (2 wppt) and 2 weeks after secondary infection with E. caproni (2 wpsi). Scale bar: 10 μm. [file 13071_2020_4467_MOESM3_ESM.tif]

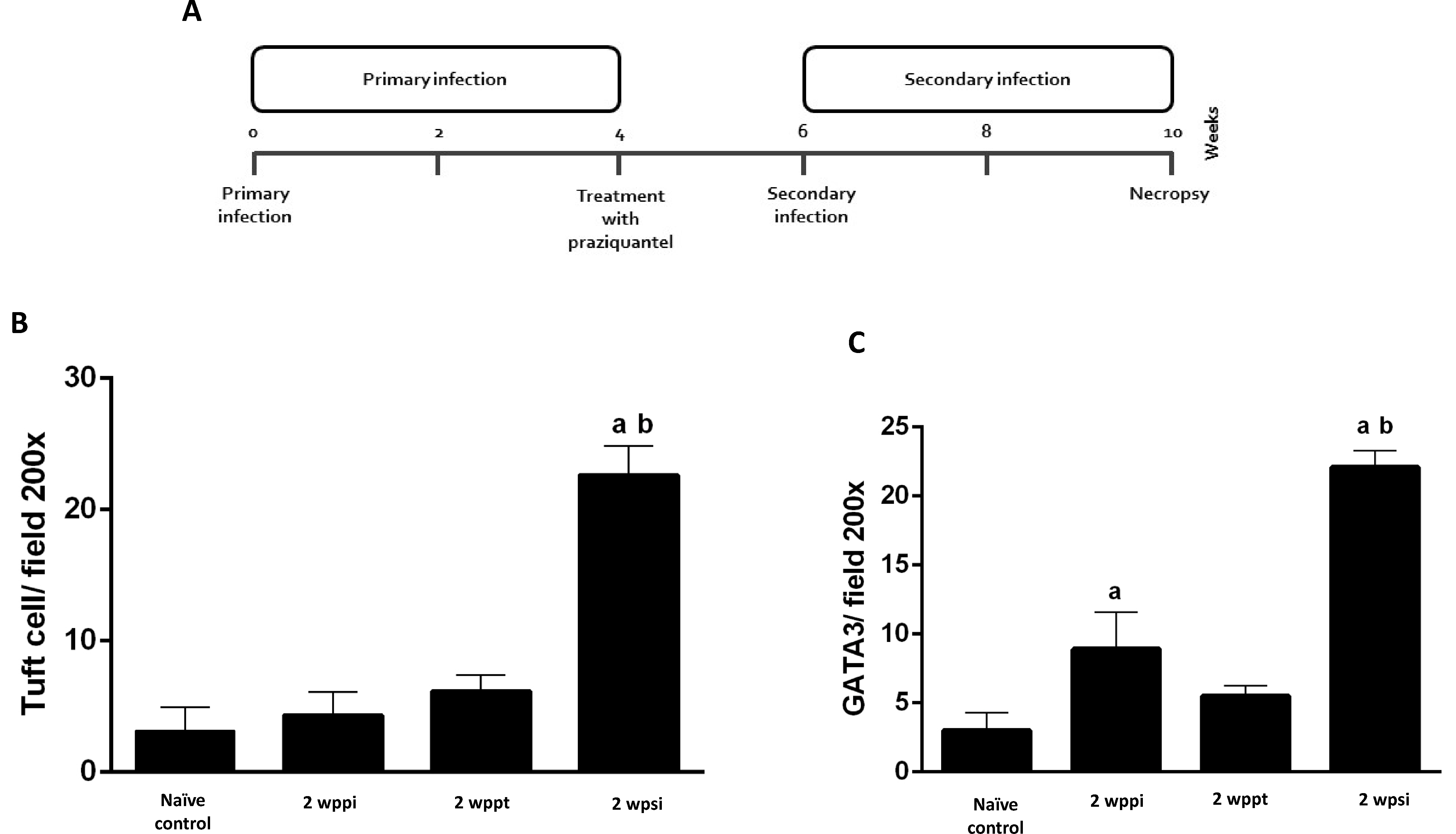

Supplement: Supplementary file 4 — Additional file 4: Fig. S3. Secondary E. caproni infection induces expansion of tuft cells and GATA3 + cells. (A) Schematic representation of the experimental protocol; (B) Counts of tuft cell populations and (C) GATA3 + cells 2 weeks after primary infection (2 wppi), 2 weeks after treatment with pzq (2 wppt) and 2 weeks after secondary infection with E. caproni (2 wpsi). Vertical bars represent the standard deviation. a: significant differences with respect to naïve mice controls; b: significant differences between groups (p < 0.05). [file 13071_2020_4467_MOESM4_ESM.tif]

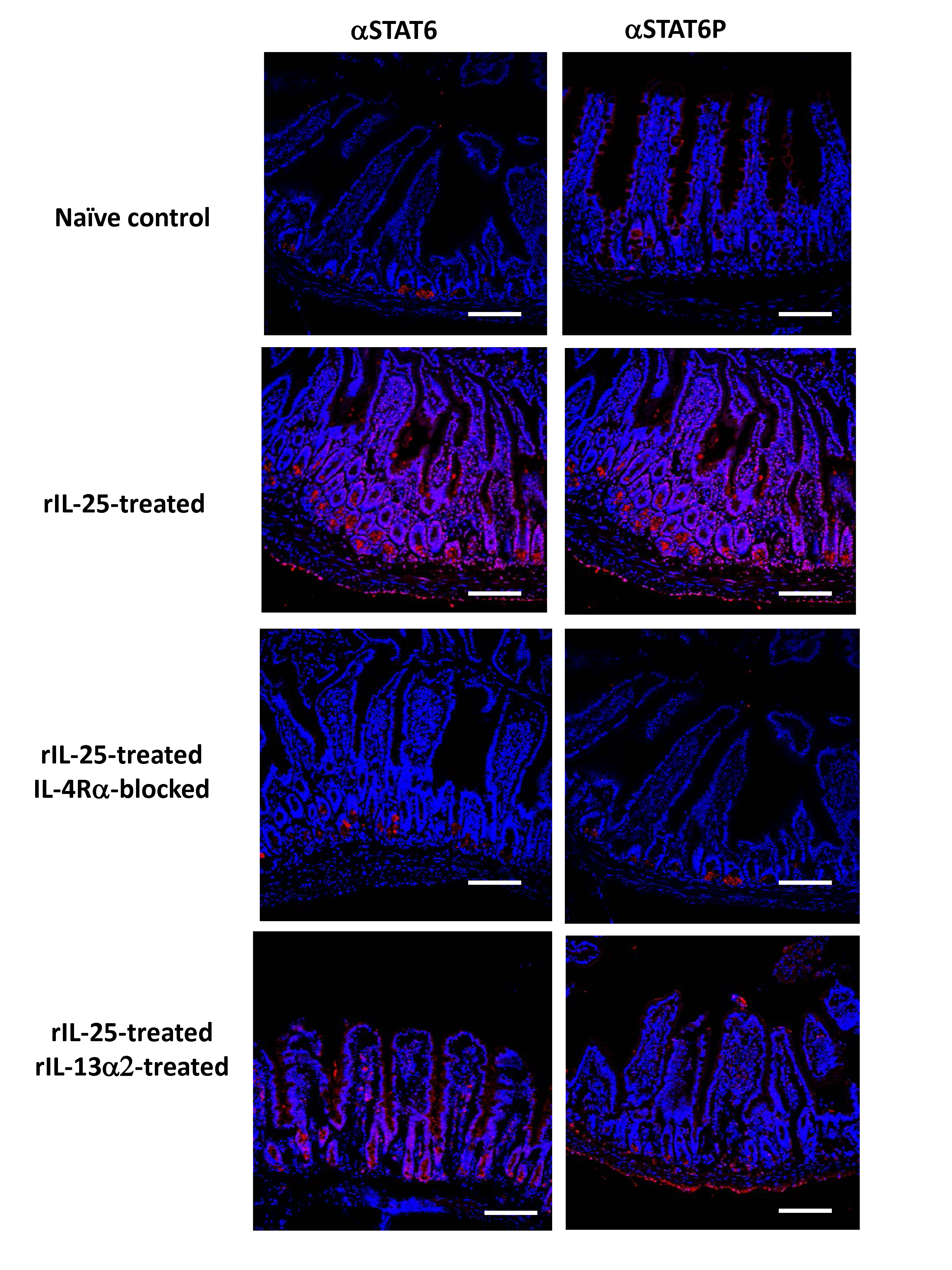

Supplement: Supplementary file 5 — Additional file 5: Fig. S4. Treatment of IL-25-treated-mice with mα-IL-4Rα or rIL-13Rα2 reduces STAT6 activation. Indirect immunofluorescence with anti-STAT6 (red) and anti-STAT6P (red) on intestinal tissue of IL-25-treated-mice that were also treated with mα-IL-4Rα or rIL-13Rα2 at 2 weeks post-primary infection. Scale bar: 30 μm. [file 13071_2020_4467_MOESM5_ESM.tif]
